# Muscularity Concerns and Disordered Eating Symptoms in Adult Women: A Network Analysis

**DOI:** 10.1002/erv.3192

**Published:** 2025-03-17

**Authors:** Vanessa Jürgensen, Georg Halbeisen, Martin S. Lehe, Georgios Paslakis

**Affiliations:** ^1^ University Clinic for Psychosomatic Medicine and Psychotherapy, Medical Faculty, Campus East‐Westphalia Ruhr‐University Bochum Lübbecke Germany; ^2^ Department of Clinical Psychology and Psychotherapy Otto‐Friedrich‐University of Bamberg Bamberg Germany

**Keywords:** body dysmorphia, drive for muscularity, eating disorders, psychotherapy, thinness ideal

## Abstract

This study examined the role of muscularity concerns in eating disorder (ED) symptoms among a sample of women. We expanded on previous research by exploring a broader range of ED symptoms, including orthorexia (ON) and avoidant/restrictive food intake disorder (ARFID). Using network analysis, we analysed data from 308 adult women (18 years or older) who completed muscularity, disordered eating, and sociodemographic assessments. Our findings revealed five interconnected symptom communities reflecting traditional ED symptoms, such as eating concerns and shape and weight overvaluation. Notably, muscularity concerns emerged as a distinct community, emphasising their relevance to ED symptoms in women. Additionally, we identified selective eating tendencies and compulsive healthy eating. Highly central symptoms were rumination about healthy eating, fear and guilt over unhealthy eating, body‐related embarrassment, and muscularity concerns (wishing to be heavier, wishing for heavier arms). These results suggest that muscularity concerns could constitute a uniquely identifiable and central diagnostic target for body image concerns and disordered eating in women.

**Summary**

- Achieving a muscular body is growing concern among women.
- We examined how muscularity concerns relate disordered eating in network analysis.
- Symptoms clustered into eating concerns and shape and weight overvaluation.
- Muscularity concerns emerged as a distinct symptom community.
- Wishing for a stronger upper body and arms had the largest influence.

## 1 Introduction

Eating disorders (EDs) are characterised by body image concerns, disturbances in eating, and weight‐control behaviours (American Psychiatric Association 2013). Globally, up to 8.4% of women and 2.2% of men develop EDs during their lifetime, including Anorexia Nervosa and Bulimia Nervosa, with Binge‐Eating Disorder and OSFED (Other Specified Feeding or Eating Disorders) accounting for the majority of cases (Galmiche et al. 2019; Santomauro et al. 2021). EDs have been placed among the most severe mental disorders in terms of psychological burden and mortality (Treasure et al. 2020). Given their severe consequences and low onset age (Solmi et al. 2022), it is essential to deepen our understanding of disordered eating presentation and to enhance early ED detection and people's access to care.

The overevaluation of body weight and shape are considered core symptoms underlying the development and maintenance of extreme dieting and weight control behaviours (Fairburn et al. 2003). Historically, these symptoms have been conceptualised in reference to a thin body ideal (Byrne et al. 2023; Halbeisen et al. 2022), which especially women in Western countries continue to internalise through media portrayals and social media use (Mingoia et al. 2017; Paterna et al. 2021). However, there is an increasing recognition that the preeminence of the thin ideal could delay the detection of disordered eating across gender and cultural groups (Halbeisen et al. 2022; Murray et al. 2017) and that body ideals and concerns among women, too, are increasingly diverse (McComb and Mills 2022). Identifying new pathways toward and characteristics of disordered eating in light of changing social norms and cultural pressures is thus an important research goal.

Media depictions of women's ideal bodies increasingly focus on visible muscularity, including of the upper body, the abdomen, and the lower body (Jerónimo and Carraça 2022). While this push toward fitness and athleticism (#fitspiration) often occurs within the context of promoting a healthy lifestyle through exercise and conscious eating (Vandenbosch et al. 2022), exposure to the “fitspiration” imagery consistently leads to stronger internalisation of muscular body ideals, a greater tendency for appearance comparisons, and increased body dissatisfaction across various genders (Jerónimo and Carraça 2022). Given that appearance‐related comparisons to internalised body ideals are important precursors of the development of disordered eating (Donovan et al. 2020), striving towards muscularity may constitute a uniquely identifiable and central diagnostic target in for disordered eating and weight control behaviours in women, too (Cunningham et al. 2022).

An increasingly popular approach for analysing disordered eating presentation and the centrality of symptoms is network analysis (Borsboom et al. 2021; Monteleone and Cascino 2021; Sala et al. 2023). In network analysis, specific symptoms are represented as *nodes*, and the interaction between symptoms as *edges* (e.g., pairwise partial correlations). The network's arrangement and topology can be evaluated to detect communities of highly interconnected symptoms, similar to factors in factor analyses, and to quantify how strongly individual symptoms influence others, which is a way of assessing a symptom's centrality. Network analysis thus provides insights into the structure of disordered eating presentation, including the relative importance of muscularity concerns, as recently shown for men (Eschrich et al. 2024).

Network analyses of disordered eating symptoms among women have largely confirmed the expected central roles of the overvaluation of body shape and weight as well as of weight loss desire (Monteleone and Cascino 2021). However, only three network analyses thus far included muscularity concerns (Tomei et al. 2022). Forbush et al. (2016) examined disordered eating symptoms in a mixed‐gender community sample at risk for EDs (77% women). The analysis identified a community of symptoms related to excessive exercising and the desire for increased muscularity in addition to restrictive dieting and body dissatisfaction communities; however, the centrality of muscularity concerns was low. DuBois et al. (2017) obtained similar results in a mixed‐gender sample of treatment‐seeking individuals diagnosed with an ED (88% women). A “muscle building” score had the lowest centrality in the overall network but was the second most central symptom in patients with Binge‐Eating Disorder. Finally, Prnjak et al. (2021) found a symptom network of body image disturbances (weight/shape overvaluation), leanness concerns (i.e., the desire to have low body fat and toned muscles), and two separate muscularity‐related attitudes and behaviours communities in a mixed‐gender sample of adolescents (53% women). Central symptoms connecting communities (called *bridge nodes*) were “traditional” symptoms related to body image disturbances (hating one's body) and leanness (striving for toned muscles), but also positive attitudes toward gaining muscles and weightlifting behaviour, suggesting a potential role of muscularity concerns in women.

However, the extent to which these network results are reproducible requires further investigation. Network results highly depend on the included variables and the type of measures used to assess these variables (Monteleone 2024; Monteleone and Cascino 2021). Detected communities and centrality estimates can change when including hitherto unaccounted symptoms (e.g., by removing spurious correlations; Prnjak et al. 2021) or when using measures that conceptualise EDs in more general symptoms (Sala et al. 2023). Thus far, network analyses with women that included muscularity concerns did so only in addition to thinness‐ or leanness‐related measures, which may have contributed to the low centrality of muscularity concerns. As we have argued elsewhere (Eschrich et al. 2024), other concerns, such as the obsessive preoccupation with healthy eating (Orthorexia Nervosa, ON), could independently account for both restrictive eating (Atchison and Zickgraf 2022) and muscle‐building behaviours (MacPhail and Oberle 2022), and should thus be included in women's symptom networks. Some women may also restrict eating based on sensory sensitivities or eating‐related anxieties, which has been described as Avoidant/Restrictive Food Intake Disorder (ARFID; American Psychiatric Association 2013). To the best of our knowledge, no study has yet explored muscularity concerns in the presence of ARFID in women's symptom networks, and their inclusion could thus further illuminate the structure of disordered eating symptoms.

It is further important to consider that several of the network analyses described above that addressed muscularity concerns in women relied on mixed‐gender samples. Network estimates do not account for group‐level correlations between muscularity concerns and other disordered eating symptoms and could thus be biased in mixed‐gender samples. For example, men could be overall more concerned with muscularity and less with food restriction compared to women, leading to an overall correlation, but without these symptoms correlating within gender groups. Indeed, Prnjak et al. (2021) found muscularity concerns in women to be less connected to other body image disturbances when compared to men, suggesting a need to investigate muscularity concerns in disordered eating networks in women separately.

### 1.1 The Present Study

This study primarily aimed to corroborate and extend previous findings on disordered eating presentation in women by examining the role of muscularity concerns among an extended range of disordered eating symptoms using network analysis. Specifically, we wanted to examine how muscularity concerns and which specific symptoms link to disturbed eating patterns, body image disturbances, and weight‐control behaviours in women, among which we included ON and ARFID symptoms for the first time (note that we did not additionally include leanness concerns due to the unavailability of a validated German measure, see Discussion). While we expected overall positive associations between the included disordered eating symptoms and muscularity concerns, we had no a priori predictions concerning the centrality of individual symptoms in the network structure.

To further inform the development of screening and assessment tools, our secondary aim was to compare ED symptoms and networks between women with low and high risk for having an ED, similar to previous endeavours (Eschrich et al. 2024). Identifying unique symptom communities, and bridge nodes connecting these communities, may help to formulate diagnostic targets for ED assessment and screening tools. As network topologies might change as a part of transitioning from a healthy to a disordered state (and vice versa), it is important to compare network structures between low and high‐risk populations. While we expected women with an increased risk for an ED to show pronounced disordered eating symptoms and body image concerns, previous findings gave no clear indication of how other symptoms or network structures could differ. Our investigation thus partly remained exploratory.

## 2 Methods

### 2.1 Participants

Similar to previous studies (Eschrich et al. 2024), we aimed to include *N* = 300 participants following sample size recommendations for estimating cross‐sectional networks (Constantin and Cramer 2019). We aimed to include adults only (aged 18 years or older), of female gender, living in Germany, who were willing to provide informed consent. We excluded participants indicating male gender, and who failed to pass the attention check (see below). We recruited participants via Prolific (www.prolific.com), on June 3rd, 2024, and among university forums and acquaintances, in May and June 2024. The study received ethics approval (AZ 2022‐910_1, March 15th, 2024), was registered at https://doi.org/10.17605/OSF.IO/EFN4V, and carried out in accordance with the Declaration of Helsinki. All participants gave informed consent. Participants recruited via Prolific received £4.50 for their participation. Eligible university students received course credit as compensation. We report all measures and exclusions. The data and materials can be obtained from the corresponding author upon reasonable request.

### 2.2 Measures

#### 2.2.1 Sociodemographic Variables

As part of an extended sociodemographic assessment to improve diversity in research (Halbeisen et al. 2022; Stadler et al. 2023), participants self‐reported their age, weight, height, regular medications, gender (male, female, diverse, no answer), sex assigned at birth, sexual orientation (attraction toward men, women, men and women, other genders not listed, no answer), German language ability (native, fluent, basic), migration background (yes, no), self‐identification as part of an ethnic minority or racialised group (yes, no, unknown), years of education (< 12 years, ≥ 12 years), marital status (single, married, divorced, widowed), living circumstances (alone, with others), subjective health condition (very good, good, fair, poor, very poor, no answer), disability (yes, no, no answer), providing care‐work (yes, no, no answer), history of eating disorders (open‐ended), ongoing ED treatment (open‐ended), subjective socioeconomic status (SES; on a scale from 1 to 10; Hoebel et al. 2015), and previous study participation (yes vs. no). The latter question served to exclude datasets from repeated participations. We also included an attention check question at random in the survey (“Please mark the word giraffe” from a series of options) to identify and exclude datasets from inattentive participants.

#### 2.2.2 Eating Disorder Risk

To stratify the sample based on ED risk, participants completed the German short version of the Eating Attitudes Test (EAT‐8; Richter et al. 2016). The EAT‐8 includes eight yes‐or‐no questions regarding core ED symptoms, with “yes” sum scores ≥ 3 serving as a conservative cut‐off value for women (Kuder‐Richardson's KR‐20 = 0.79).

#### 2.2.3 Symptom Assessment

##### 2.2.3.1 Disordered Eating

As in previous studies (Eschrich et al. 2024; Forrest et al. 2019; Prnjak et al. 2021), we used the validated German version of the Eating Disorder Examination‐Questionnaire (EDE‐Q; Fairburn and Beglin 1994; Hilbert et al. 2007). The EDE‐Q assessed cognitive and behavioural ED symptoms within the last 28 days along four subscales, “(Dietary) Restraint”, “Eating Concern”, “Shape Concern”, and “Weight Concern”, using 22 attitudinal items rated on a seven‐point scale (from 0, never, to 6, every day). Six additional items assessed overeating episodes, binge episodes, binge days, and purging behaviours, that is, vomiting, laxative use, and driven exercise. Since previous investigations did not support the proposed factor structure of the EDE‐Q (Laskowski et al. 2023), we reported only the global mean score across attitudinal items (Cronbach's *α* = 0.96).

The Duesseldorf Orthorexia Scale (DOS; Barthels et al. 2015) captured ON, the fixation on healthy nutrition and associated behaviours, over the previous 7 days with 10 statements (e.g., I prioritise healthy eating over pleasure) rated on a four‐point scale (from 1, does not apply at all, to 4, fully applies). We report the scale mean across all items (Cronbach's *α* = 0.87).

The Eating Disorders in Youth‐Questionnaire (EDY‐Q; van Dyck and Hilbert 2016), adapted for adults and the only available German ARFID questionnaire (Hilbert et al. 2021), assessed ARFID symptoms using 14 questions covering food avoidance (FA; *α* = 0.37), selective eating (SE, *α* = 0.73), functional dysphagia (FD, *α* = 0.77), and problems with underweight. Additional questions assessing pica, rumination, and weight/shape concerns (ARFID exclusion criteria) are not used for scale construction. All items were rated on a seven‐point scale (from 0, never, to 6, always). We report the EDY‐Q subscale and total mean scores (Cronbach's *α* = 0.61).

##### 2.2.3.2 Muscularity Concerns

The validated German version of the Muscle Dysmorphic Disorder Inventory (MDDI; Zeeck et al. 2018) included 13 items on muscularity‐related body image disturbances and behaviours rated on a 5‐point scale (from 1, never, to 5, always), including the subscales drive for size (DS, e.g., body too skinny; *α* = 0.81), appearance intolerance (AI, e.g., hate my body; *α* = 0.84), and functional impairment (FI, e.g., depressed if not exercising; *α* = 0.84). We report the subscale and MDDI total means (Cronbach's *α* = 0.78).

The Female Body Scale (FBS) and Female Fit Body Scale (FFITBS) are figural rating scales designed to measure participants' body dissatisfaction regarding muscularity (BD‐M) and thinness, that is, having low body fat (BD‐F; Ralph‐Nearman and Filik 2020). The FBS depicted a series of nine female bodies ranging from emaciated to obese, and the FFITBS a series of nine female bodies ranging from gaunt to very muscular. For each series of bodies, women were asked to indicate which body figure best represented their current body and then to indicate their ideal body, with actual‐ideal discrepancy scores serving as the primary outcomes.

#### 2.2.4 Additional Questions

Further questions, not covered in this report, assessed therapy motivation, social contacts, autistic traits, the perception of stigma toward individuals with EDs, and gender‐role self‐concepts (Jürgensen et al., Lehe et al., Laskowski et al., unpublished).

### 2.3 Procedure

We implemented the online study on our web server using jsPsych (de Leeuw et al. 2023). Upon assessing the study's website, participants read the study information and consent forms. We explained that the study concerned EDs in women and that participants could optionally receive feedback on their ED risk. After providing informed consent, participants completed the sociodemographic questionnaire and the ED risk assessment. The remaining questionnaires were completed next, in randomised order. If participants consented to receive feedback, the EAT‐8 score and an explanatory text were displayed. If participants did not consent to receive feedback, they directly advanced to the last screen and were thanked and dismissed. We estimated the study to be completed in 15–20 min.

### 2.4 Data Analysis

We closely followed the analytic procedure outlined in Eschrich et al. (2024). That is, we initially characterised and compared ED and associated symptom profiles between women with low and high ED risk, using the validated scales' overall and subscale scores. We used independent samples *t*‐tests when comparing continuous variables, Pearson correlations for associations, and Chi‐squared (χ^2^) frequency tests when comparing categorical variables, with a significance threshold of *p* < 0.05, in SPSS 28 (IBM Corp 2021). We screened for multivariate outliers across questionnaire scores using Mahalanobis distance with a criterion of *p* < 0.001 to detect irregularities in the data (e.g., from people just clicking through the questionnaires) and examined minimum completion times before analysis.

We estimated participants' symptom networks using R 4.3.3 (R Core Team 2022). Specifically, we estimated Gaussian Graphical Models (GGM) based on Pearson correlations via the *qgraph* package 1.9.8 (Epskamp et al. 2012) with glasso (graphical least absolute shrinkage and selection operator) regularisation and EBIC (Extended Bayesian Information criterion) tuning parameter selection (*γ* = 0.5), which reduces false discovery rates compared to the classical BIC (Foygel and Drton 2010). This yields a sparse, undirected network of conditional associations between included symptoms (i.e., controlled for all other symptoms in the network), with small and unstable edges set to zero (Epskamp and Fried 2018). Before estimating the network, all items were standardised based on their scale range, and missing responses (0.2% overall) were imputed using predictive mean matching via the *mice* package 3.16.0 (van Buuren and Groothuis‐Oudshoorn 2011).

**FIGURE 1:**
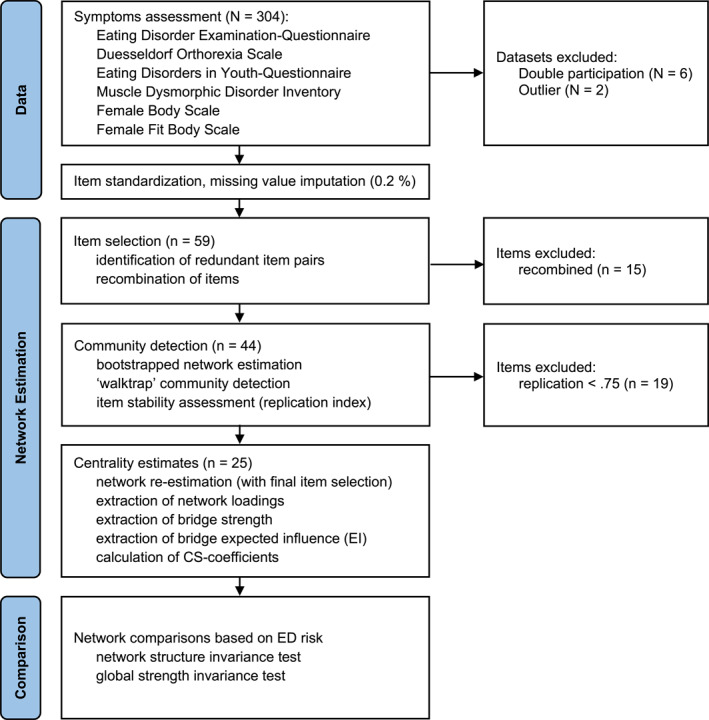
Flowchart illustrating the network analysis procedure. *N* denotes number of datasets (participants), *n* refers to the number of items included in the analysis.

The network estimation involved the following steps (Figure 1): First, we selected items to serve as network nodes from all assessments with the “goldbricker” and “net_reduce” functions in *networktools* 1.5.2 (Jones 2023). The functions identify and remove redundant item pairs (those with less than a specified threshold proportion of significantly different correlations with the remaining items), given that multicollinearity may obscure the true symptom structure. We used the default significance and proportion threshold parameters (0.05 and 0.25, respectively), and kept the first principal components of the redundant pairs as new variables (i.e., the “PCA” method, preserving information about which items were redundant in the final results).

Second, we determined symptom communities via “bootEGA” in *EGAnet* 2.0.5 (Golino and Christensen 2023; Golino and Epskamp 2017). The function derives the typical network structure from the median values of edges across 1000, non‐parametric bootstrapped samples, and then uses “Walktrap” to detect symptom communities. Using bootstrapped community detection represented a crucial improvement over previous investigations, as it empirically assessed the stability of communities and items (how often one detects a specific number of communities and replicates the items assignment across bootstrap iterations). Simulations show that items with replication rates lower than 0.75 can be considered “unstable” (Christensen and Golino 2021b), which we therefore removed before proceeding.

Third, we determined centrality estimates for the average (bootstrapped) network of the final item selection. Going beyond previous studies, we computed two types of symptom centrality estimates within and between communities: network loadings and bridge centrality. Network loading, extracted from the average network using “net.loads” in *EGAnet*, index the sum of connections of a node to other nodes of the same community; bridge strength and bridge expected influence, the recommended bridge centrality indices extracted via “bridge” in *networktools*, instead of index connections between nodes of different communities (Jones et al. 2021). Bridge strength is the sum of absolute edge values, which reflects the extent to which one node influences nodes from other communities. Bridge expected influence (EI) is the sum of positive and negative edges, which thus reflects the overall direction of influence of one node onto nodes of other communities (we did not compute node strength or node expected influence since these measures do not take the clustering of symptoms into distinct communities into account and thus potentially conflate two conceptually distinct sources of covariation). Because networks are estimated with some degree of error, we also determined the stability of centrality estimates via *bootnet* 1.6 (Epskamp et al. 2018) using *CS*‐coefficients, which quantify the proportion of cases that can be dropped to retain, with 95% certainty, a correlation with the original centrality parameters higher than 0.70. To interpret centrality parameters, *CS*‐coefficients should not be below 0.25, and preferably above 0.5. We also used the *bootnet* package's non‐parametric, bootstrapped difference test to evaluate differences in centrality parameters.

Finally, we used *NetworkComparisonTest* 2.2.2 (van Borkulo et al. 2022) to compare symptom networks between women with high and low ED risk. The permutation‐based hypothesis test used the selected nodes and communities from the overall network (see above) and assessed the difference between estimated networks in the high‐ and low‐risk groups. The tests provide several invariance measures, evaluated against a random sampling of groups from the datasets, of which we examined network structure invariance (which evaluates if all edges are equal) and global strength invariance (which evaluates if the overall level of connectivity is the same across risk groups).

## 3 Results

### 3.1 Sample Characteristics

We recruited *N* = 304 female participants in total; after checking the inclusion criteria and removing two multivariate outliers, *N* = 296 cases were analysed (288 women, 6 diverse, 2 no answer; *M*_age_ = 28.44, age range: 18–64 years). Completion times ranged from 7.75 to 107 min (*M* = 23.09, SD = 11.80). The EAT‐8 classified *n* = 85 as low risk for having an ED and *n* = 211 as high risk. The risk groups were of similar age, *p* = 0.71. However, higher ED risk was associated with an increased body mass index (BMI), *p* < 0.001, lower subjective SES, *p* < 0.001, and lower general health, *p* = 0.003. Other demographics were not associated with ED risk (see Table 1).

**TABLE 1:** Sample sociodemographic.

Parameter	Total	High risk	Low risk	*p*‐value
*n*	296	211	85	
Age (years)	28.4 (9.5)	28.6 (8.7)	28.1 (11.4)	0.71
BMI (kg/m^2^)	23.51 (5.2)	24.5 (5.6)	21.0 (2.7)	< 0.001
Gender				0.53
female	288	204	84	
diverse	6	5	1	
no comment	2	2	0	
Sexual preference				0.10
women	24	22	2	
men	192	132	60	
women & men	67	49	18	
other	2	2	0	
no comment	11	6	5	
German Language				0.21
first language	245	170	75	
fluent	45	37	8	
basic knowledge	6	4	2	
Education				0.38
less Than 12 years	47	36	11	
12 years or more	249	175	74	
Migration background				0.06
yes	90	71	19	
no	206	144	66	
Part of minority group				0.07
yes	26	21	5	
no	257	140	66	
“I don't know”	15	14	1	
Marital status				0.22
married/partnership	58	45	13	
single	229	176	79	
divorced	9	8	1	
Living situation				0.81
alone	78	56	22	
with others	217	154	63	
no comment	1	1	0	
Health status				0.003
very good	61	34	27	
good	159	114	45	
medium	66	56	10	
poor	9	7	2	
no comment	1	0	1	
Current ED treatment				0.67
yes	20	16	4	
no	169	128	41	
*no response*	107	67	40	
Disability				0.79
yes	13	9	4	
no	281	201	80	
no comment	2	1	1	
Subjective SES	5.9 (1.5)	5.8 (1.6)	6.5 (1.4)	< 0.001

*Note:* High and low risk groups based on EAT‐8 sum scores (≥ 3 for high risk, < 3 for low risk). *p*‐values denote group differences based on independent sample *t*‐test (for continuous variables) and χ^2^ test (for categorical variables).

### 3.2 Symptom Profiles

Table 2 displays the total and risk groups' means, and comparison statistics. Consistent with the screening categorisation, the high‐risk group presented with higher EDE‐Q, DOS, and MDDI global scores than the low‐risk group. However, of the MDDI subscales, only functional impairment (FI) and appearance intolerance (AI) were elevated, but not drive for size (DS). Similarly, women with high risk showed pronounced thinness discrepancies (BD‐F) and even reduced muscularity discrepancies (BD‐M). EDY‐Q food avoidance was elevated in the high‐risk group; EDY‐Q total and subscale scores did not differ.

**TABLE 2:** Questionnaire means (standard deviations in parentheses) and comparisons.

Variable	Total (*N* = 296)	High risk (*n* = 211)	Low risk (*n* = 85)	*t*	*p*	*d*
EDE‐Q	1.92 (1.39)	2.46 (1.28)	0.56 (0.37)	13.50	< 0.001	1.74
DOS	1.89 (0.54)	2.03 (0.53)	1.54 (0.41)	7.69	< 0.001	0.99
EDY‐Q	1.27 (0.70)	1.27 (0.68)	1.25 (0.76)	0.19	0.85	0.02
FA	1.48 (1.00)	1.58 (1.01)	1.24 (0.94)	2.67	0.008	0.34
SE	1.89 (1.49)	1.94 (1.49)	1.75 (1.48)	0.97	0.33	0.13
FD	0.38 (0.83)	0.41 (0.88)	0.28 (0.71)	1.30	0.20	0.17
MDDI	2.01 (0.55)	2.13 (0.54)	1.71 (0.47)	6.38	< 0.001	0.82
DS	1.80 (0.74)	1.77 (0.74)	1.89 (0.75)	−1.30	0.20	−0.17
AI	2.59 (1.01)	2.94 (0.94)	1.71 (0.52)	11.29	< 0.001	1.45
FI	1.70 (0.78)	1.79 (0.81)	1.47 (0.65)	3.19	0.002	0.41
BD‐F	1.09 (1.09)	1.40 (1.05)	0.32 (0.74)	8.70	< 0.001	1.12
BD‐M	−0.34 (1.23)	−0.57 (1.30)	0.21 (0.82)	−5.15	< 0.001	−0.66

*Note:* High and low risk groups based on EAT‐8 sum scores (≥3 for high risk, < 3 for low risk).

Abbrievation: AI, appearance intolerance; BD‐F, body fat dissatisfaction; BD‐M, muscularity dissatisfaction; DOS, duesseldorf orthorexia scale; DS, drive for size; EDE‐Q, eating disorder examination‐questionnaire; EDY‐Q, eating disorders in youth‐questionnaire; FA, food avoidance, FD, functional dysphagia; FI, functional impairment; MDDI, muscle dysmorphic disorder inventory; SE, selective eating.

### 3.3 Symptom Network

#### 3.3.1 Item Selection

We included all scale‐building items of the EDE‐Q (plus binge episodes and urge to exercise), DOS, MDDI, EDY‐Q, and the discrepancy scores of the muscularity and body fat‐related body dissatisfaction (we excluded EDE‐Q´s laxative abuse and self‐induced vomiting as fewer than 5% of participants reported these behaviours; similar to Eschrich et al. (2024), we coded binge episodes as missing prior to imputation in case they exceeded the reported number of overeating episodes). Goldbricker recombined 15 pairs of the original 59 items, leaving 44 (combined) items. bootEGA identified another 19 items with replication indices lower than 0.75. After removing these, we ended up with a final selection of 25 for the remaining analyses (11 EDE‐Q [3 combined, 1 combined with MDDI], 6 DOS [1 combined]; 5 MDDI [3 combined, plus 1 combined with EDEQ and 1 combined with EDY‐Q]; 3 EDY‐Q [1 combined with MDDI]; and BD‐M [but not BD‐F]).

#### 3.3.2 Community Detection

**FIGURE 2:**
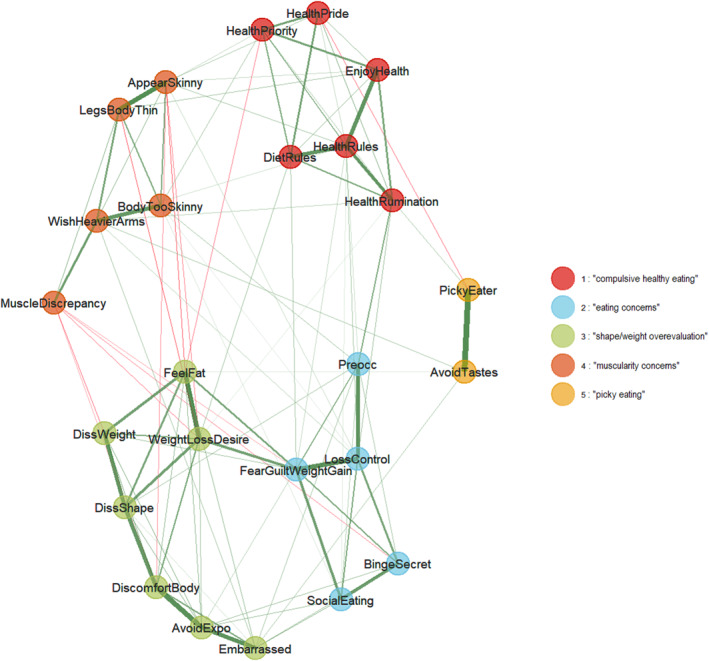
The average network of eating disorder symptoms in adult women. Thicker lines represent stronger edges (regularised conditional correlations), with green indicating positive and red negative edges. The colours of the nodes indicate empirically determined symptom communities.

Figure 2 shows the median network. The items consistently formed five symptom communities in 95% of cases across 1000 bootstrap samples, with all item replication indices ≥ 0.95. *Community 1* included items of the DOS related to obsessive healthy eating and rumination as well as dietary rules and boundaries. *Community 2* included EDE‐Q items related to fear and guilt related to weight gain and eating anxiety in social situations as well as binge episodes. *Community 3* included EDE‐Q and MDDI items that addressed body dissatisfaction, and body concealment and discomfort. *Community 4* captured the MDDI's “drive for size” subscale, muscularity‐related body image discrepancies (BD‐M), and the EDY‐Q's underweight concerns. Finally, *Community 5* included the (combined) items of the EDY‐Q's selective eating subscale.

#### 3.3.3 Centrality Estimates

**FIGURE 3:**
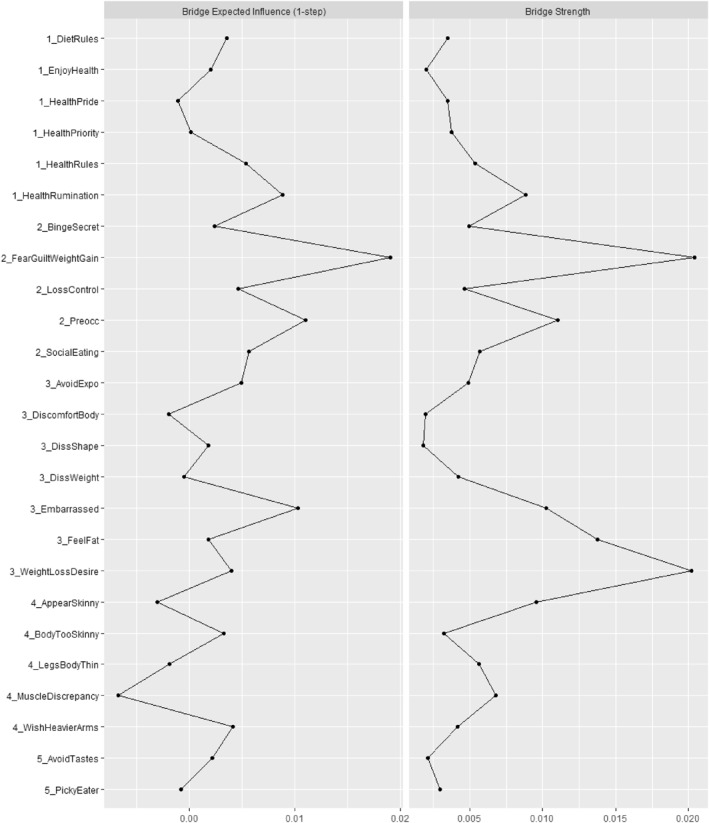
Normalised bridge centralities values ordered by symptom community. Bridge strength is the sum of absolute edge values, which reflects the extent to which one node influences nodes from other communities; bridge expected influence (EI) is the sum of positive and negative edges, which thus reflects the overall direction of influence of one node onto nodes of other communities.

Table 3 shows the network loadings (within‐community centrality), and Figure 3 the bridge (between‐community) centrality estimates. The most central (i.e., highest‐loading) symptoms within communities 1 to 5 were health rules, that is, having difficulties breaking dietary rules and avoiding friends that eat unhealthy (*Community 1*), fear of losing control over eating (*Community 2*), discomfort seeing one's body (*Community 3*), thinking that legs are too slender and wishing to be heavier (*Community 4*), and avoiding food with specific smells, appearances, or tastes (*Community 5*).

**TABLE 3:** Network loadings of items across communities.

Variables	Scale IDs	Description	Com1	Com2	Com3	Com4	Com5
Health Rules	DOS 9 + DOS 4	Difficulty going against dietary rules + avoiding unhealthy friends	0.46				
Diet Rules	DOS 2	Adherence to nutritional rules	0.34				
Enjoy Health	DOS 3	Enjoying only healthy foods	0.33				
HealthRuminaton	DOS 8	Thoughts revolve around healthy nutrition	0.29				
HealthyPriority	DOS 1	Health more important than enjoyment	0.26				
HealthyPride	DOS 5	Liking to pay more attention to healthy nutrition	0.22				
LossControl	EDE‐Q 9	Fear of losing control over eating		0.46			
FearGuiltWeightGain	EDE‐Q 20 + EDE‐Q 10	Guilt about eating + fear of weight gain		0.37			
SocialEating	EDE‐Q 21	Social eating		0.27			
BingeSecret	EDE‐Q binge + EDE‐Q 19	binge episodes + eating in secret		0.25			
Preocc	EDE‐Q 8 + EDE‐Q 7	Preoccupation with shape or weight + preoccupation with food, eating, calories		0.23			
DisscomfortBody	EDE‐Q 27	Discomfort seeing body			0.38		
DissShape	EDE‐Q 26	Dissatisfation with shape			0.37		
AvoidExpo	EDE‐Q 28	Avoidance of exposure			0.35		
FeelFat	EDE‐Q 11	Feeling of fatness			0.33		
WeightLossDesire	MDDI 7 + EDE‐Q 12	Feeling of too much bodyfat + desire to lose weight			0.32		
DissWeight	EDE‐Q 25	Dissatisfaction with weight			0.30		
Embarrassed	MDDI 9 + MDDI 2	Embarrassed without shirt + wearing loose clothing			0.19		
LegsBodyThin	MDDI 6 + EDY 5	Feeling legs too thin + wishing to have more weigh				0.39	
WishHeavierArms	MDDI 8 + MDDI 4	Wishing arms stronger + wishing to be heavier				0.37	
Appear skinny	EDY 4	Others think too skinny				0.32	
BodyTooSkinny	MDDI 5 + MDDI 1	Finding chest too small + thinking body is too slender				0.27	
MuscleDiscrepancy	BD‐M	Muscularity perceived/desired discrepancy				0.15	
AvoidTastes	EDY 12	Avoid foods for taste, consistency, looks, or smell					0.42
PickyEater	EDY 9 + EDY 8	Avoid trying new foods + being picky					0.42

*Note:* Network loadings represent each node's (unique) contribution to the emergence of a symptom community.

The most important symptoms in terms of absolute influence on other communities (bridge strength) were health rumination, that is, thoughts constantly revolving around healthy eating (*Community 1*), guilt about eating and fear of weight gain (*Community 2*), weight loss desire (*Community 3*), appearing skinny (*Community 4*), and being a picky eater (*Community 5)*. Comparison tests showed that only bridge strength for fear and guilt of weight gain and being a picky eater significantly differed from each other (Supporting Information Figure S1). No other bridge strength estimates differed significantly from the others. The most important symptoms in terms of directional influence, bridge EI, remained largely comparable to bridge strength, albeit with two notable exceptions: In *Community 3*, feeling embarrassed had the largest net positive effect on symptoms of other communities, whereas the influence of weight loss desire was markedly reduced. Similarly, in *Community 4,* appearing too skinny and muscle‐related dissatisfaction remained import, but their net effect turned negative. Instead, wishing for a stronger upper body and arms had the largest net positive effect on symptoms of other communities. Thus, neither weight loss desire nor striving for muscle mass were associated with an overall activation of the symptom network. Comparison tests showed that bridge EI for fear of and guilt related to weight gain differed significantly from health rumination, avoiding tastes, and also the wish for stronger arms (see Supporting Information Figure S2). *CS*‐coefficients were consistently larger than 0.50, suggesting that the centrality estimates were interpretable (0.75 for edge strength, 0.74 for bridge EI and 0.64 bridge strength, respectively; because CS‐coefficients for network loadings are not available, we report edge strength, which combines network and bridge strength loadings; Christensen and Golino 2021a).

#### 3.3.4 Network Comparisons

Finally, we compared symptom networks between women with high and low ED risk using the nodes and communities from the overall network. Between risk groups, both the network structure invariance test and the global strength invariance test were significant, *M* = 0.38, *p* = 0.02 and *S* = 4.57, *p* < 0.01, respectively. Thus, there were some indications that edges differed and that symptoms were more connected in the high‐risk compared to the low‐risk group. The centrality estimates in the high‐risk group were acceptably stable (0.65 for edge strength, 0.43 for bridge EI, and 0.35 for bridge strength). However, when attempting to calculate centrality estimates for the low‐risk groups with the default estimation parameters, no significant edges were found and an empty network was returned as the best fitting model. Thus, we were unable to report stability estimates for the low‐risk group and suggest to interpret the network comparisons with caution (indeed, the comparison tests were nonsignificant when using the EAT‐8 median to define high‐ and low‐risk groups, *M* = 0.23, *p* = 0.64 and *S* = 1.17, *p* = 0.12, with acceptable [> 0.56] and moderate [> 0.31] centrality estimates, respectively).

## 4 Discussion

Previous network analyses suggested that muscularity concerns may play a significant role in ED symptoms, concerning all gender and ethnic groups, though uncertainty remained about their reproducibility when accounting for previously unexamined symptoms and focussing on an exclusively female sample. To address this, we aimed to replicate and expand on these findings by exploring the role of muscularity concerns across a broader range of ED symptoms, including ON (via the DOS) and ARFID (via the EDY‐Q).

Consistent with our hypothesis of overall positive associations among the included disordered eating symptoms, the empirically selected items from validated measures formed five symptom communities, similar to a previous study in men (Eschrich et al. 2024). The communities included muscularity concerns and core aspects of eating disorder psychopathology (Fairburn et al. 2003), with the specific patterns largely corroborating previous findings. Similar to Forrest et al. (2019), *Community 2* encompassed eating concerns, that is, the preoccupation with food, calories, and body weight or shape, and “ED‐specific forms of fear and avoidance”, including the fear of losing control over eating, anxiety about being observed while eating, and fear and guilt related to weight gain. Binge‐eating episodes also fell within this community, consistent with the idea of binge eating as a coping mechanism (Dalton 2024). Fear and guilt exerted the greatest influence on other communities, emphasising that self‐directed negative emotions may constitute a universal diagnostic target (Levinson and Williams 2020).

Similarly, *Community 3* emerged as shape and weight overvaluation. Shape concerns, feeling too fat, wanting to lose weight, and avoiding exposure of one's body, were closely linked to shame and body dissatisfaction. This community thus reflected a “traditional” view of female disordered eating presentation, consistent with mixed‐gender sample network analyses (Wang et al. 2019). It is interesting to note that some previous studies also included the fear of weight gain among body dissatisfaction communities (which our analysis assigned to *Community 2*), which may have stemmed from a previous lack of differentiating between dissatisfaction with weight and dissatisfaction with body shape among the included items in the network analysis (Prnjak et al. 2021). This calls for a more nuanced understanding of the different components related to body image. Further emphasising the critical role of negative emotionality for disordered eating, embarrassment emerged as this community's most import bridge node in terms of a net‐positive effect.

Most importantly, we also identified muscularity concerns as a further symptom community (Forbush et al. 2016; Prnjak et al. 2021). Specifically, *Community 4* reflected a desire for a more muscular physique, particularly regarding the upper body and extremities, and discrepancies in muscle‐related body image (BD‐M). These characteristics align with the notion that a muscular body can be highly valued among women as well. Some items of *Community 4*, for example, discrepancies in muscle‐related body image, expectedly stood apart from thinness‐related items in *Communities 2* and *3*, and deactivated nodes in these communities. This could suggest that muscularity concerns in women are more subtle and also aligns with the findings of Holland et al. (2014), who demonstrated that unhealthy physical activity manifests differently in men and women, with more compulsive and compensatory qualities found in women and more excessive training to improve visible muscle mass found in men. Future research should further investigate these differences to better understand how muscle building needs and perceptions differ between genders.

Extending previous findings, we also found a community reflective of compulsive healthy eating (*Community 1*). The orthorexic phenotype of this community included obsessive thoughts about healthy eating and adhering to a strict food routine. While the updated diagnostic criteria have recognised EDs such as OSFED and ARFID (Silén and Keski‐Rahkonen 2022), the nosological status and clinical relevance of ON remains an issue of ongoing discussion (Meule and Voderholzer 2021). However, our network showed rumination about healthy nutrition to bridge to other disordered eating communities (see also Eschrich et al. 2024), suggesting some aspects of orthorexic eating as a potential risk factor for other disordered eating symptoms.

Finally, our network also covered selective eating tendencies, that is, picky eating behaviours such as avoiding certain smells, tastes, or food appearances, and refusing to try new foods. Although these symptoms were linked weakly within the network, their presence further demonstrates that “picky eating” is not limited to early childhood and may emerge later in life (Lange et al. 2019). Considering ARFID symptoms could thus help to identify disordered eating in women.

Taken together, these findings may underscore the importance for clinical research and practice to consider the diversifying pathways toward disordered eating in light of changing social norms and cultural pressures. Although the thin ideal still remains prominent (which is also reflected in our network), social media trends increasingly promote muscular, lean, but also “voluptuous” body ideals for women as well (Belmonte et al. 2024; Vandenbosch et al. 2022). Such shifts task researchers to identify similarities in the underlying mechanisms leading to disordered eating (Rodgers et al. 2024), and to construct equivalence classes of eating and body shaping concerns and behaviours that occur within the context of diverse body ideals. As stated above, identifying symptom communities and bridge nodes in network analyses may help to formulate these classes and to eventually adjust diagnostic targets and ED criteria. Meanwhile, clinicians may take these and similar findings as an opportunity to reflect on their own perceptions and assumptions of people with EDs and acknowledge the potential of biases in preventing people from accessing care (Halbeisen et al. 2022; Lehe et al. 2024; Sonneville and Lipson 2018). Disordered eating may come in all shapes and sizes, and potentially in the guise of (alleged) health‐oriented behaviours, and thus clinicians should remain vigilant.

### 4.1 Limitations

While the present results may help to enhance our understanding of disordered eating presentation in women, we must acknowledge several limitations. One major limitation is its reliance on correlational data, making it difficult to establish causal relationships between symptoms. Additionally, network analysis may not fully capture the complexities of individual experiences, as the results are often shaped by the input data used and the decisions made beforehand. For example, although we included measures related to thinness and muscularity, we did not additionally measure leanness concerns, that is, the desire for having low body fat *and* toned muscles. Some data suggests that the drive for leanness could be associated with less maladaptive behaviours compared to the drives for thinness and muscularity (Lang and Rancourt 2020), whereas others found leanness to account for unique variance in disordered eating and exercise behaviours (Cunningham et al. 2022). There are currently no validated questionnaires for assessing the drive for leanness in German. If available, leanness‐related measures should be included in future network investigations to potentially reveal additional disordered eating‐related symptom communities (Prnjak et al. 2021). In a similar vein, future studies should also include more general psychopathological symptoms which may emerge as bridge nodes between disordered eating symptoms or highlight important “external” conditions that can lead to or protect against disordered eating symptoms (Monteleone 2024).

Furthermore, we must note several limitations inherent to our sampling. Our study utilised a non‐clinical sample, leaving it unclear whether muscularity concerns play a similar central role among women with clinically diagnosed EDs and those in treatment. Although we assessed self‐reports of ED symptoms, risk, and previous treatment history, we did not include face‐to‐face evaluations that would be required to derive a clinical diagnosis. Another limitation is that more than half of the participants were classified as “at risk”, which may have affected the results. It is possible that our convenience sample was biased, as participants may have been motivated to take part due to high personal interest in the study's topic. We also cannot exclude that the EAT‐8 might have been prone toward high positive rates (cf. similar rates in Pieh et al. 2021). Despite an additional comparison of groups based on the EAT‐8 median showing no group differences (see Results), future studies should aim to include similar numbers of high‐ and low‐risk individuals to allow for sufficiently powered comparisons. It is further important to note that participants completed the study online and remotely, potentially reducing data precision and leading to more selective samples. Data collection on online survey platforms is usually associated with a higher proportion of low‐quality responses (Douglas et al. 2023), especially for participants using study participation as a main source of income (Peer et al. 2022). In fact, part of our sample was incentivised to participate through monetary compensation, potentially creating a less representative sample. Although additional network comparison tests based on compensation showed no significant differences in terms of structure (*M* = 0.25, *p* = 0.52) or global strength (*S* = 0.78, *p* = 0.41), it would be important to replicate the present findings with a more representative sampling approach.

Finally, we must acknowledge that our results and analysis do not account for the influence of sociodemographic factors in disordered eating presentation (Halbeisen et al. 2022; Traut et al. 2023). Our sample primarily included single, *cis*‐gender, heterosexual women, leaving little room for meaningful comparative analyses. However, past research consistently showed that adults and adolescents in sexual and gender minority populations tend to be at greater risk for both subclinical disordered eating and eating disorders diagnoses when compared to their heterosexual and cisgender counterparts (Parker and Harriger 2020). Disordered eating incidence has also increased in recent years among underrepresented ethnic groups (Cheng et al. 2019; Rodgers et al. 2018). These socio‐demographical shifts point to a critical role of personal, social, and cultural identity in disordered eating aetiology and to potential variations in disordered eating symptom associations. For example, peer pressures and body dissatisfaction appear to be pronounced in lesbian and bisexual women (Hazzard et al. 2019), suggesting that certain communities could be more prone to reinforcing societal expectations regarding ideal body types. Whether and how disordered eating network structures could differ between sociodemographic groups should thus be further explored.

## 5 Conclusions

Despite the limitations, our findings provide several important conclusions. Muscularity concerns emerged as one cluster within women's disordered eating networks, particularly in relation to body‐shaping goals. Thus, current screenings and diagnostic tools widely used in women may need to be revised accordingly to capture these aspects, and new equivalence classes of diverse eating and body shaping concerns and behaviours may need to be constructed. Moreover, symptom bridges appeared along affective dimensions, especially the fear of weight gain, the guilt about eating, and being embarrassed about the own body, suggesting that items related to strong emotions play a role in linking communities. Given that behaviours and ideals are influenced by sociocultural norms and changes, understanding the burden associated with these behaviours may be especially useful for detecting potential cases of EDs among women.

## Conflicts of Interest

The authors declare no conflicts of interest..

## Supporting information

Supporting Information

## Data Availability

The data that support the findings of this study are available from the corresponding author upon reasonable request.
