# Supplementary material for: Muscularity Concerns and Disordered Eating Symptoms in Adult Women: A Network Analysis
Source: Eur Eat Disord Rev. 2025 Mar 17;33(5):864–78. doi: 10.1002/erv.3192 (PMC12319133; doi:10.1002/erv.3192)
Supplement: Supplementary file 1 — Supporting Information [file ERV-33-864-s001.docx]

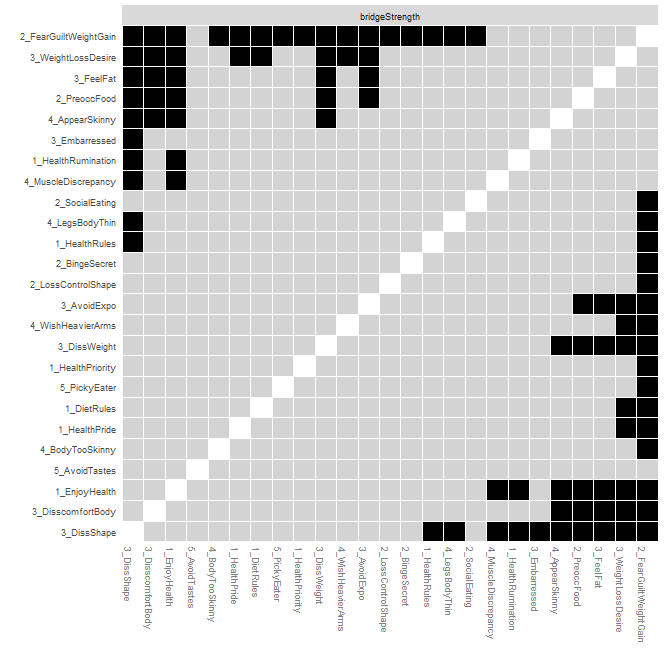
***Figure S1***. Results of bridge centrality difference tests. Black boxes indicate significant differences between edges in terms of centrality; gray boxes indicate nonsignificant differences.


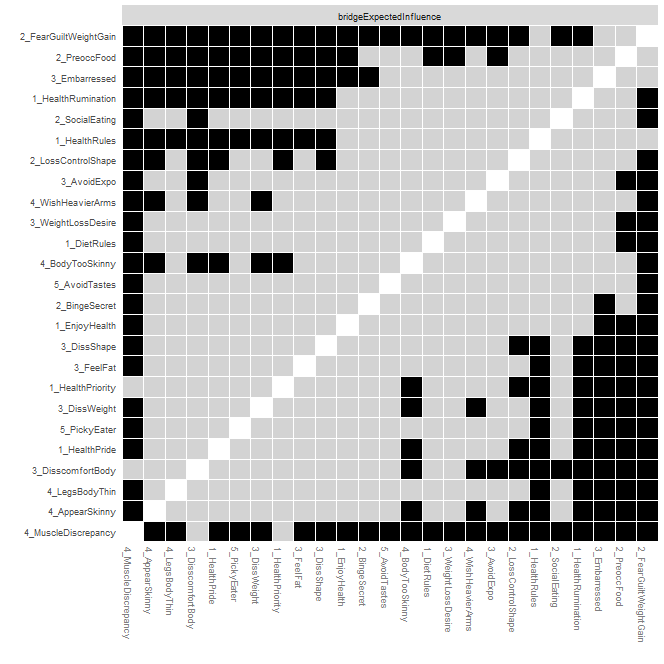
***Figure S2***. Results of bridge EI centrality difference tests. Black boxes indicate significant differences between edges in terms of centrality; gray boxes indicate nonsignificant differences.
